# Identification of structurally closely related monosaccharide and disaccharide isomers by PMP labeling in conjunction with IM-MS/MS

**DOI:** 10.1038/srep28079

**Published:** 2016-06-16

**Authors:** Hongmei Yang, Lei Shi, Xiaoyu Zhuang, Rui Su, Debin Wan, Fengrui Song, Jinying Li, Shuying Liu

**Affiliations:** 1 Changchun University of Chinese Medicine, Changchun 130117, China; 2 Changchun Institute of Applied Chemistry, Chinese Academy of Sciences, 5625 Renmin Street, Changchun 130022, China; 3 High Temperature Reactor Holdings Co., Ltd., China Nuclear Engineering Group Co., Beijing 100037, China; 4 Department of Entomology and Comprehensive Cancer Center, University of California, Davis, CA 95616, United States

## Abstract

It remains particularly difficult for gaining unambiguous information on anomer, linkage, and position isomers of oligosaccharides using conventional mass spectrometry (MS) methods. In our laboratory, an ion mobility (IM) shift strategy was employed to improve confidence in the identification of structurally closely related disaccharide and monosaccharide isomers using IMMS. Higher separation between structural isomers was achieved using 1-phenyl-3-methyl-5-pyrazolone (PMP) derivatization in comparison with phenylhydrazine (PHN) derivatization. Furthermore, the combination of pre-IM fragmentation of PMP derivatives provided sufficient resolution to separate the isomers not resolved in the IMMS. To chart the structural variation observed in IMMS, the collision cross sections (CCSs) for the corresponding ions were measured. We analyzed nine disaccharide and three monosaccharide isomers that differ in composition, linkages, or configuration. Our data show that coexisting carbohydrate isomers can be identified by the PMP labeling technique in conjunction with ion-mobility separation and tandem mass spectrometry. The practical application of this rapid and effective method that requires only small amounts of sample is demonstrated by the successful analysis of water-soluble ginseng extract. This demonstrated the potential of this method to measure a variety of heterogeneous sample mixtures, which may have an important impact on the field of glycomics.

Carbohydrates play critical roles in a large number of biological processes such as protein conformation, molecular recognition, and cellular interaction^1^,^2^,^3^. While their structural elucidation is an essential prerequisite for understanding their many functions at the molecular level, the diversity of the constituent monosaccharides, anomeric configuration, and glycosidic linkages makes this task analytically demanding^4^,^5^. This is one reason why glycomics lags behind the advances in genomics and proteomics. Moreover, due to the difficulty in the separation and purification of carbohydrates, the preparation from biological sources is frequently accompanied by complex mixtures, where isomers must be distinguished in order to achieve complete identification.

Many strategies have been employed for analysis of carbohydrates, such as NMR spectroscopy^6^ and high-performance liquid chromatography (HPLC)^7^, often with the goal of recognizing isomers. The NMR-based approach is efficient for evaluating isomeric heterogeneity, and for structural elucidation, but has the limitation of needing considerable amounts of the analytes and obtaining single molecular species. In comparison, HPLC is time-consuming, and unambiguous identification of isomers is often not possible. Mass spectrometry (MS) plays an important role in structural elucidation of carbohydrates due to its high sensitivity and analysis speed^8^,^9^,^10^,^11^,^12^,^13^. However, analysis of carbohydrates by MS has been challenging in part due to the frequent presence of large amounts of oligosaccharide isomers, which often display very similar collision-induced dissociation (CID) mass spectra.

Ion mobility mass spectrometry (IMMS) is a unique gas phase ion separation technique on the basis of parameters such as collision cross section (CCS), charge, mass, drift gas polarizability and lifetimes of ion-neutral gaseous complexes^14^,^15^. IMMS is a promising approach to overcoming the above mentioned limitations, making it an ideal candidate for differentiation of isomers. The Synapt G2 high definition mass spectrometry (HDMS)^16^,^17^, traveling wave ion mobility mass spectrometry (TWIMMS)^18^,^19^, is a hybrid quadrupole/ion mobility separator/orthogonal time-of-flight (TOF) MS instrument. More recently, IMMS has been increasingly applied to the separation and analysis of small molecules and biomacromolecules in the gas phase based on measuring their arrival time distributions (ATD) and their CCSs^20^,^21^,^22^,^23^. TWIMMS has been applied in the field of carbohydrate research, and has been reported to unambiguously distinguish both simple standards and biological mixtures of isomeric oligosaccharides^24^,^25^,^26^,^27^,^28^,^29^,^30^,^31^,^32^,^33^. Identifying oligosaccharides by TWIMMS was demonstrated^24^,^25^,^26^,^27^ by both pre- and/or post-IM fragmentation prior to MS analysis, enhancing confidence in carbohydrate identification. In most of the cases, the separation of carbohydrates by IMMS has been performed for the sodiated precursor ions in the positive mode. However, the ionic radii, valence of cations, and number of metal ion adducts will distinctly affect the conformation and separation of carbohydrate isomers in IMMS^28^,^29^,^30^. In addition, better separation among oligosaccharide isomers can be achieved in the negative ion mode^31^,^32^,^33^,^34^,^35^, with or without addition of anion salts. However, it was recently demonstrated that compositional isomeric carbohydrates could not be differentiated by IMMS in the recent article in *Nature*^33^. Harvey *et al*.^34^ reported the use of TWIMMS combined with negative ion fragmentation, for determining the structures of high-mannose glycans. Analysis of high-mannose N-glycans by TWIMMS revealed the presence of distinctive gas-phase conformers exclusive to [M–H]^−^ ions^35^. Isomer separation of small carbohydrates by IMMS has also been reported^22^,^36^, but the analytes are not fully resolved. To increase the CCSs of the oligosaccharide isomers, Fenn and McLean^37^ have employed boronic acid derivatization of carbohydrates as an ion mobility shift strategy, but no arrival time distributions of the derivatized isomers were reported.

Recently, Both *et al*.^38^ reported IMMS separation of isobaric monosaccharides and differentiation of CID fragment ions from disaccharides and polysaccharides, yet not all isomers were distinguishable. 1-Phenyl-3-methyl-5-pyrazolone (PMP), initially reported as a labeling reagent for reducing carbohydrates by Honda’s group^39^, has been widely used for derivatization of reducing carbohydrates because the derivitization is fast, mild, and has a simple clean-up procedure. Here, we present a novel method using PMP derivatization followed by IMMS for the simultaneous structural analysis of carbohydrate isomers. In an effort to obtain better ion mobility separation, we investigated factors including wave velocities, wave heights, and derivatization reagents. Water-soluble ginseng monosaccharides (WGOS-1) and water-soluble ginseng disaccharides (WGOS-2) were used to evaluate this method, demonstrating the powerful applicability of this approach for analysis of mixtures.

## Results and Discussion

### Arrival time distributions (ATDs) of 10 disaccharide and 4 monosaccharide isomers

**Figure 1:**
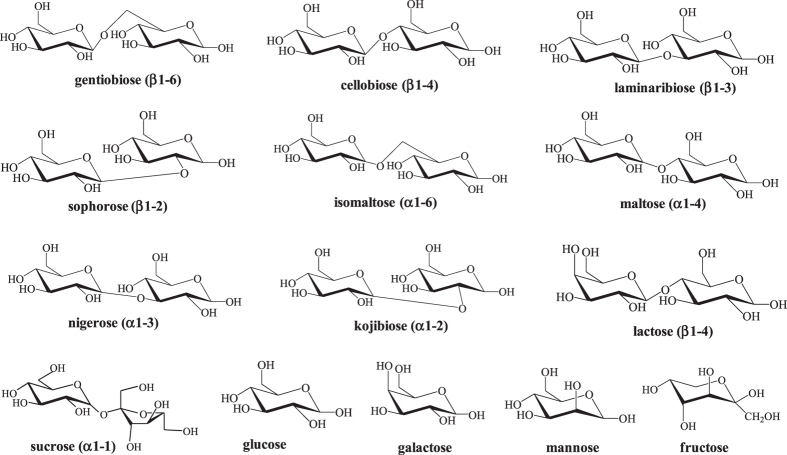
Structures for the 10 disaccharides and 4 monosaccharides used in this study.

The structures of all the 10 disaccharides and 4 monosaccharides are shown in Fig. 1. Among them, it is noteworthy that the eight closely related structural isomers including gentiobiose, cellobiose, laminaribiose, sophorose, isomaltose, maltose, nigerose, and kojibiose are all glucopyranosyl-glucose disaccharides. In the case of the monosaccharides, three epimers (glucose, galactose, and mannose) differing only in their stereochemistry, and a ketose(fructose), were selected as models. The selected carbohydrates differ only in linkages (such as gentiobiose (β1-6) and cellobiose (β1-4)), configurations (such as gentiobiose (β1-6) and isomaltose (α1-6)), and composition (such as galactose and mannose). Thus, they are difficult to be distinguished from each other. In order to increase the efficacy of ion mobility at segregating the coexisting isomers, the major experimental parameters affecting TWIM separation, traveling wave velocity and height, were optimized. The overall ion mobility spectra of the 10 disaccharide isomers and 4 monosaccharide isomers at a 40 V, 550–2500 m/s T-Wave are displayed in Supplementary Fig. S1. In all spectra, the predominant carbohydrate ions produced by electrospray ionization (ESI) were found to be the Na^+^ adducts of the disaccharides (*m/z* 365.11) and monosaccharides (*m/z* 203.05). Ammonia-adducted ions were also detected at low abundance for the four monosaccharide structural isomers; however, their drift times are exactly the same under any experimental condition (data not shown).

As observed in Supplementary Fig. S1, the drift times of sucrose and the other disaccharides differ by at least 0.11 ms; those of glucose and its isomers differ by 0.11 ms. Consequently, sucrose and glucose were more readily distinguished with their respective isomers by their drift times. However, the drift times of the rest of the studied saccharides were essentially indistinguishable. These results for the monosaccharide isomers were in agreement with the previous report^38^.

### Effect of derivatization reagents

**Figure 2:**
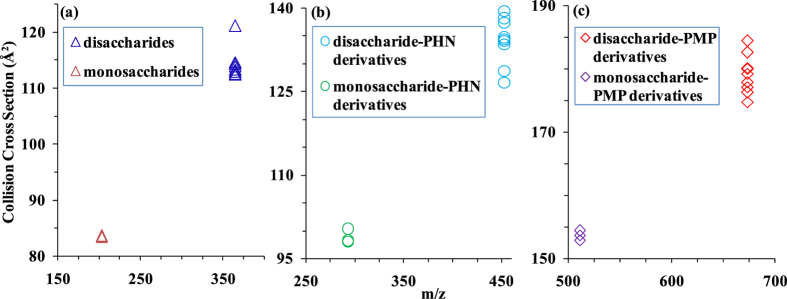
A plot of CCS vs. *m/z* for (**a**) [M + Na]^+^ of underivatized carbohydrates, and (**b**) [M + Na]^+^ of PHN- and (**c**) [M + H]^+^ of PMP-derivatized carbohydrate isomers. RE (relative error) <5%. Refer to Table 1 for tabulated values.

The ion mobility shift reagent strategy was employed to increase the CCSs of the above mentioned disaccharides and monosaccharides, with the aim of distinguishing the isomers. Two commonly used derivatization reagents of carbohydrates, PMP and PHN, were used to covalently modify saccharides. The generalized schemes for the reaction of carbohydrates with PMP and PHN were shown in Supplementary Fig. S2, with cellobiose used as an example. The mass shifts resulting from carbohydrate derivatization with PMP and PHN are 330 and 88 Da, respectively (Supplementary Fig. S3). The CCSs for the underivatized and derivatized species were calculated to determine the effect of derivatization on the resulting structural shift in IMMS analysis. For the PMP-derivatized species, the preponderance (99%) of the signal was for protonated species, so sodium-coordinated CCSs were not reported for comparison. The effect of derivatization on the CCSs of carbohydrates is illustrated in Fig. 2. The desired shift in conformation space was achieved for most species, as indicated by a greater increase in CCSs. Our data clearly demonstrate that carbohydrate isomers with identical mass but different conformation can be partly distinguished based on the CCSs of their derivatives.[Table t1]

**Figure 3:**
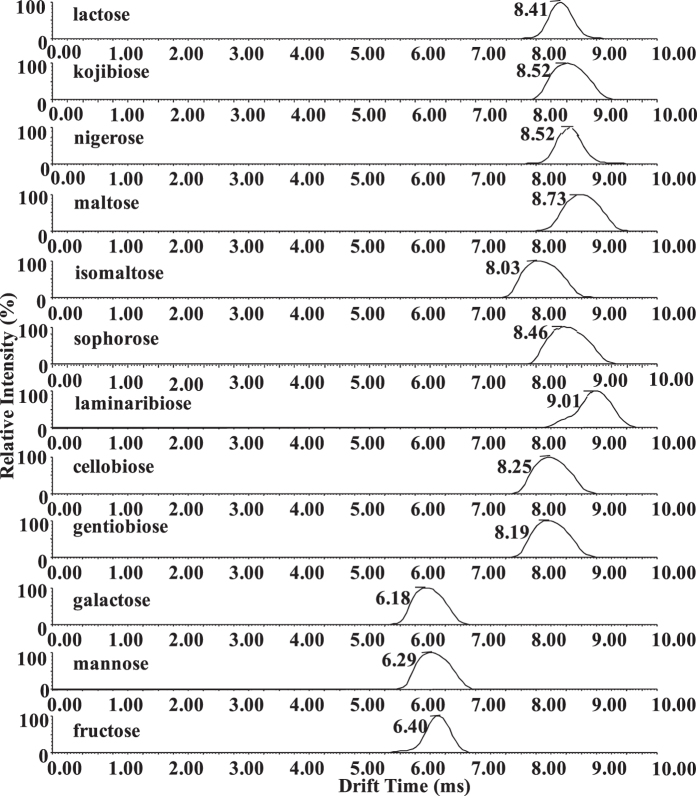
Overall mobility spectra of the 9 PMP-derivatized disaccharide isomers and 3 PMP-derivatized monosaccharide isomers. All mobility spectra of the disaccharides and monosaccharides were extracted for protonated ions at *m/z* 673.27 and 511.22, respectively. The arrival times are from three individual measurements, and deviation is ± 0.01 ms.

As expected, the saccharides showed different drift times by the ion mobility shift reagent strategy. As illustrated by the ATDs presented in Fig. 3, the three disaccharide isomers maltose (t_D_ = 8.73 ms), isomaltose (t_D_ = 8.03 ms), and laminaribiose (t_D_ = 9.01 ms) as well as the three monosaccharide isomers galactose (t_D_ = 6.18 ms), mannose (t_D_ = 6.29 ms), and fructose (t_D_ = 6.4 ms) were more readily distinguished by the drift times of their PMP derivatives. The leading edge (i.e. laminaribiose) may arise due to the bisPMP binding conformations since the laminaribiose standard and monoPMP-laminaribiose contain only a single isomer (Supplementary Fig. S4). Unfortunately, the drift times of three groups of disaccharide isomers were essentially indistinguishable as the PMP derivatives (t_D_ = 8.41 ms for [PMP-lactose + H]^+^ versus t_D_ = 8.46 ms for [PMP-sophorose + H]^+^; t_D_ = 8.52 ms for [PMP-kojibiose + H]^+^ versus t_D_ = 8.52 ms for [PMP-nigerose + H]^+^; t_D_ = 8.25 ms for [PMP-cellobiose + H]^+^ versus t_D_ = 8.19 ms for [PMP-gentiobiose + H]^+^). In the case of the monosaccharide isomers, better separation was acquired, and PMP-fructose (t_D_ = 6.40 ms) had longer drift times than PMP-mannose (t_D_ = 6.29 ms) and PMP-galactose (t_D_ = 6.18 ms). Similar results were obtained for PHN derivatization (Supplementary Fig. S5).

Overall the presence of leading/training peaks was more prevalent with PHN labeling (Supplementary Fig. S5), indicating multiple conformations potentially arising due to differential sites of sodium-adduction associated with these structures in the gas phase^38^. In contrast, PMP-disaccharide peaks were broader (Fig. 3), due to the larger molecular weight of the compounds. We noted that peak width was related to the size of the compound instead of the derivatization. For example, underivatized maltotetraose has a similar molecular weight compared to the PMP derivatives of disaccharides, and also had very broad peak width (Supplementary Fig. S6). This observation was in good agreement with a previous report^40^. Interestingly, the width of ATDs varied significantly for some of the different disaccharide derivatives (e.g. lactose versus kojibiose, Fig. 3). The differences of the widths of ATDs for the same mass have also been observed in analysis of cyclodextrin (CD) by IM MS^40^. The explanation was that the linear sugar chains had more conformational variations than the cyclic αCD moiety having the same mass, which caused an IM peak broadening of γ–CD. Here, it was inferred that the width differences of ATDs for some disaccharides resulted from their different spatial configurations.

In short, the results showed that the majority of disaccharide and monosaccharide structural isomers exhibited unique mobility drift times, even though not all of them were fully resolved.

### Tandem mass spectrometric analysis of PMP derivatives of disaccharides

**Figure 4:**
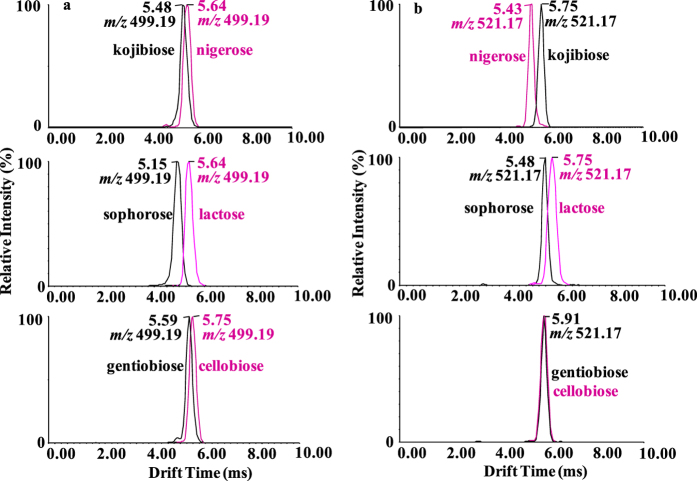
IM-MS/MS of the protonated PMP derivative ions of the three groups of disaccharide isomers. (**a**) The mobility spectra were extracted for protonated product ions at *m/z* 499.19. (**b**) The mobility spectra were extracted for sodiated product ions at *m/z* 521.17. The arrival times are from three individual measurements, and deviation is ± 0.01 ms.

The MS^2^ strategy was utilized to further enhance the identification of specific disaccharides. The mobility and mass selected ions could be introduced into the trap cell installed in front of a TWIMS, which enables the isomeric heterogeneity of product ions to be evaluated. The product ions in the tandem mass spectra of PHN derivatives of disaccharides (Supplementary Fig. S3e) were the corresponding native disaccharide ions. Thus, it was impossible to differentiate the six unresolved disaccharide isomers mentioned above by the mobility spectra extracted for the product ions at *m/z* 365.11, as shown in Supplementary Fig. S1. To solve this problem, we took recourse to MS^2^ analysis of PMP derivatives of disaccharides (Fig. 4). The mobilities of the product ions, [monoPMP-disaccharide + H]^+^ ions at *m/z* 499.19 (Fig. 4, panel a) and [monoPMP-disaccharide + Na]^+^ ions at *m/z* 521.17 (Fig. 4, panel b), were examined and compared. The only difference between the two specific ions is the ionized form. From Fig. 4, it is clearly seen that the ionized form exerted a significant influence on the drift times of the product ions. As a general trend, drift times of these ions increased as the size of the ions increased. For example, the drift time of kojibiose varied from t_D_ = 4.83 ms as the [M + H]^+^ ion to t_D_ = 5.75 ms for the [M + Na]^+^ ion.

As presented in Fig. 4a, it became immediately apparent that the product ions of the three pairs of isomers exhibited strikingly different drift times (t_D_ = 5.48 ms for [monoPMP-kojibiose + H]^+^ versus t_D_ = 5.64 ms for [monoPMP-nigerose + H]^+^; t_D_ = 5.64 ms for [monoPMP-lactose + H]^+^ versus t_D_ = 5.15 ms for [monoPMP-sophoros + H]^+^; t_D_ = 5.75 ms for [monoPMP-cellobiose + H]^+^ versus t_D_ = 5.59 ms for [monoPMP-gentiobiose + H]^+^)), depending on differences in their linkages. Whereas, as shown in Fig. 4b, some monoPMP disaccharide isomers were separable in the form of sodium ion adducts (different t_D_ values) while not in the other (same t_D_ values), which indicated that sodium ion adducts are often not the preferred charge carrier from the standpoint of IM separation of isomeric carbohydrates. Sodium ion association was disadvantage to the differentiation of the monoPMP derivatives of the linkage isomers cellobiose (β1-4) and gentiobiose (β1-6). In comparison, better separation among structural isomers appeared to be achieved for [M + H]^+^ cations. The CCSs for the twelve MS^2^ fragment ions were calculated to determine the structural variation (Supplementary Table S1). The sodiated monoPMP derivatives of the linkage isomers cellobiose (β1-4) and gentiobiose (β1-6) exhibited CCSs that were almost identical to each other (151.52 Å^2^ and 151.10 Å^2^, respectively), which is in good agreement with the results from ATDs. Remarkably, protonated monoPMP derivatives of the six disaccharide isomers exhibited highly diagnostic CCS values that differed by at least 2.1 Å^2^ for each pair of isomers.

**Figure 5:**
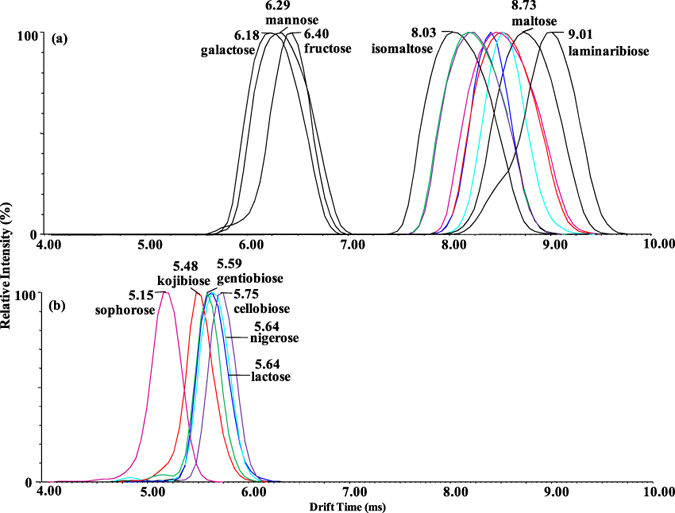
(**a**) Overall mobility spectra of the mixture of 9 PMP-derivatized disaccharide isomers and 3 PMP-derivatized monosaccharide isomers. All mobility spectra of the disaccharides and monosaccharides were extracted for protonated ions at *m/z* 673.27 and 511.22, respectively. (**b**) IM-MS/MS of the six unresolved PMP-disaccharide derivatives (kojibiose, nigerose, lactose, sophoros, cellobiose, and gentiobiose) unlabelled in the upper spectra. The mobility spectra were extracted for the product ions at *m/z* 499.19.

The mixture of the given set of disaccharide and monosaccharide isomers can be differentiated by PMP derivatization in conjunction with ion-mobility separation and MS^2^. For clarity, the overlaid IMS plots of all the analytes are summerized in Fig. 5.

### Application to WGOS isolated from the *Panax Ginseng* root

**Figure 6:**
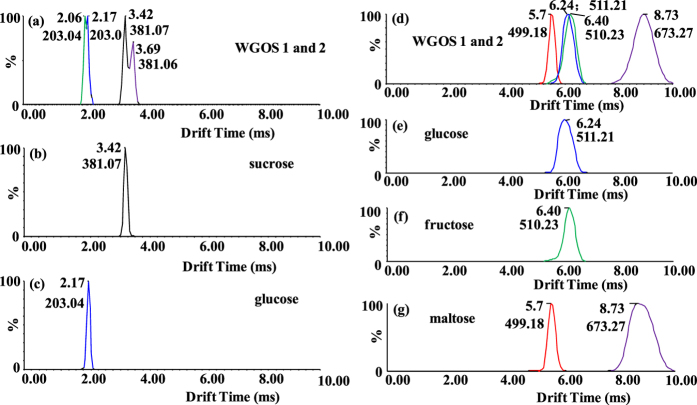
ATDs of (**a**) WGOS-1 and WGOS-2, (**b**) sucrose, and (**c**) glucose in the left column. ATDs of protonated PMP derivatives of (**d**) WGOS-1 and WGS-2, (**e**) glucose, (**f**) fructose, and (**g**) maltose in the right column. The red traces in Figure d and g are the drift time plots of fragment ions from the ions at *m/z* 673.27. The arrival times are from three individual measurements, and deviation is ± 0.01 ms.

As an example of the application of the above technique, Fig. 6 shows the IM spectra of WGOS-1 and WGOS-2 obtained from a warm-water extract of *Panax ginseng* roots as our previously published procedures^41^. Direct comparison of Fig. 6a (sodiated WGOS-1 at *m/z* 203.04 and potassium adduct ions of WGOS-2 at *m/z* 381.07), 6b (potassium adduct ions of sucrose at *m/z* 381.07), and 6c (sodiated glucose at *m/z* 203.04) revealed the presence of sucrose in WGOS-2 and glucose in WGOS-1. The peaks at 2.06 and 3.69 ms in Fig. 6a are ambiguous. The former could be assigned as fructose, mannose, or galactose based on Supplementary Fig. S1, and the later could correspond to kojibiose, nigerose, maltose, sophorose, laminaribiose, or cellobiose (Supplementary Fig. S7). CCS values of the potassium adduct ions of the disaccharide isomers were summarized in Supplementary Table S2.

In order to identify the peaks at 2.06 and 3.69 ms, PMP-derivatized WGOS-1 and WGOS-2 were determined (Fig. 6d). A comparison of the drift times between Fig. 6d,e, and f showed that WGOS-1 was composed of glucose and fructose, indicating the peak at 2.06 ms corresponded to fructose instead of mannose and galactose (Fig. 6a). Furthermore, taking these results from the tandem mass spectra and full scan mass spectra into consideration, one can conclude that WGOS-2 contains maltose rather than the other disaccharides (Figs 6d,g). Sucrose is a nonreducing disaccharide, thus its PMP derivatives was not detected.

The results showed that the disaccharides in WGOS-2 and monosaccharides in WGOS-1 were identified as sucrose and maltose as well as glucose and fructose, respectively, which were in agreement with the observations made in a previous study^42^. Thus, the reliability of this method was confirmed.

## Conclusions

The differentiation of closely related structural isomers is a serious complication when using mass spectrometry alone. In this study, the coexisting mono- and disaccharide isomers with different linkages, compositions, and configurations were separated by the PMP labeling technique in conjunction with ion-mobility separation and tandem mass spectrometry. The extent of separation was significantly affected by the ionized forms of MS^2^ fragments, and [M + H]^+^ cations are the preferred charge carrier from the standpoint of IM separation of isomeric carbohydrates. In addition, our data show that the structural differences between carbohydrate isomers can lead to distinctly different CCSs. Therefore, carbohydrate isomers can be distinguished not only on the basis of their drift time, but also based on their relative CCS values. All carbohydrates in WGOS-1 and WGOS-2 have been successfully examined using this new method. The simplicity and validity of the method makes it an attractive option for unequivocal differentiation of carbohydrate isomers.

## Material & Methods

### Chemicals and Reagents

Kojibiose, maltose, isomaltose, nigerose, cellobiose, gentiobiose, melibiose, trehalose, α-cyclodextrin, glucose, fructose, galactose, mannose, and PHN were bought from J&K Chemical Ltd. (Beijing, China). Sophorose was acquired from Shanghai Huicheng Biotechnology Co, Ltd. (Shanghai, China). Laminaribiose was acquired from Beijing Chemsynlab Pharmaceutical Science & Technology Co. Ltd. (Beijing, China). Maltotriose, raffinose, melezitose, sucrose, PMP, and lactose were acquired from Aladdin (Shanghai, China). Methanol (HPLC grade) was obtained from Fisher Chemical Company. All other chemicals used in this study were of analytical grade and were used without further purification. High-purity helium, nitrogen, and argon (99.999%) were supplied by Changchun Juyang Gas Co., Ltd. (Changchun, China). Ultrapure water (specific conductivity, 18.2 MΩ/cm) was produced by a MilliQ device (Millipore, Milford, MA, USA).

### Sample Preparation

For ion mobility studies, 2 mM stock solutions of monosaccharides and disaccharides were prepared using ESI solvent (50% aqueous methanol, v/v) and were diluted in 1:20 ESI solvent for individual analysis (100 μM each). These were stored at–20 °C until needed. NH_3_·H_2_O–methanol solution was prepared by adding 560 μL NH_3_·H_2_O (25%) to 9.44 mL methanol. The labeling reagent solution used for derivatization (250 mM) was prepared by dissolving 60.67 mg of PMP into 10 mL NH_3_·H_2_O–methanol solution. PMP and PHN derivatives of monosaccharides and disaccharides were prepared according to the previously described procedures^43^,^44^. The detailed derivatization procedures are included in the Supplementary Information.

### Mass Spectrometry and Ion Mobihlity

All mass spectrometry and ion mobility experiments were performed using a Waters Synapt G2 quadrupole-IM-TOF mass spectrometer with TWIM capabilities (Waters, Manchester, U.K.). The samples were analyzed in positive ion mode with a capillary voltage at 2.8 kV. The Synapt G2 parameters were optimized as follows: sample cone voltage at 50 V, extraction cone voltage at 4 V, source temperature at 120 °C, and desolvation temperature at 350 °C. The flow rates of the cone gas and desolvation gas were set to 30 and 450 L h^−1^, respectively.

The major experimental parameters affecting TWIM separation are the drift gas pressure, the TWIM DC traveling wave height, and the TWIM DC traveling wave velocity^45^,^46^. For all IM experiments, He was introduced at 180 mL/min to the helium cell installed in front of the ion mobility separator, and nitrogen was used as the drift gas at a flow rate of 90 mL/min. The traveling wave height was set to 40.0 V. The traveling wave velocity was systematically optimized for maximum resolution. Optimal traveling wave velocities were 550–2500 m/s (the variable IMS wave velocity, start velocity: 550 m/s, end velocity: 2500 m/s) for monosaccharides and disaccharides as well as their derivatives. IM-MS/MS experiments were performed to make an attempt to distinguish barely resolved analytes in the IM-MS. The CID experiments were performed using argon as collision gas at the trap cell of the instrument at a flow rate of 2 mL/min and a collision energy of 40 V. Data acquisition and processing were conducted using Masslynx 4.1 software (Waters Corp., Manchester, U.K.).

### Collision Cross Sections

It has been proposed that the CCS is proportional to t_D_^X^ in the traveling-wave IMS system. The exponential factor *X* depends upon many variables including the traveling wave height and the traveling wave velocity^46^,^47^. CCS calculations were performed according to previously described protocols^47^,^48^. The CCSs of the isomeric monosaccharides and disaccharides as well as their derivatives are thus determined according to the calibration curve constructed using the oligosaccharides with known CCSs (cellobiose, 112.4 Å^2^; maltose, 112.6 Å^2^; sucrose, 108.9 Å^2^; lactose, 121.1 Å^2^; melibiose, 112.2 Å^2^; trehalose, 110.6 Å^2^; maltotriose, 142.9 Å^2^; raffinose, 138.8 Å^2^; melezitose, 133.5 Å^2^; α-cyclodextrin, 200.7 Å^2^.)^28^,^29^. IMMS data of the calibrant ions and analytes were recorded over a range of wave heights and velocities to separate the ions. Under each condition, a calibration curve was established to calculate the experimental CCSs.

## Additional Information

**How to cite this article**: Yang, H. *et al*. Identification of structurally closely related monosaccharide and disaccharide isomers by PMP labeling in conjunction with IM-MS/MS. *Sci. Rep*. **6**, 28079; doi: 10.1038/srep28079 (2016).

## Supplementary Material

Supplementary Information

## Figures and Tables

**Table 1 t1:** The CCSs for underivatized, and PMP- and PHN-derivatized carbohydrate species (n = 16).

carbohydrates	collision cross section (Å^2^)		
	[M + Na]^+^	[M + PHN + Na]^+^	[M + PMP + H]^+^
lactose	121.1 ^a^(σ 6.4)	137.3 ± 0.3	177.9 ± 0.6
kojibiose	114.3 ± 0.4	134.0 ± 0.2	180.0 ± 0.5
nigerose	112.9 ± 0.5	126.5 ± 0.6	180.1 ± 0.4
maltose	112.6 ± 0.4^b^	138.1 ± 0.2	182.5 ± 0.3
isomaltose	113.3 ± 0.4	134.7 ± 0.3	174.7 ± 0.2
sophorose	114.6 ± 0.6	133.4 ± 0.7	179.2 ± 0.2
laminaribiose	113.9 ± 0.5	128.6 ± 0.5	184.4 ± 0.3
cellobiose	112.4 ± 0.4^b^	139.4 ± 0.3	177.1 ± 0.2
gentiobiose	114.7 ± 0.9	134.3 ± 0.2	176.2 ± 0.3
galactose	83.5 ± 0.3	98.2 ± 0.4	152.8 ± 0.1
mannose	83.4 ± 0.5	98.0 ± 0.5	153.7 ± 0.2
fructose	83.7 ± 0.4	100.3 ± 0.5	154.4 ± 0.2

^a^The value was reported in reference 28, and ^b^the values were reported in reference 29.
