# Supplementary material for: Identification of structurally closely related monosaccharide and disaccharide isomers by PMP labeling in conjunction with IM-MS/MS
Source: Sci Rep. 2016 Jun 16;6:28079. doi: 10.1038/srep28079 (PMC4910106; doi:10.1038/srep28079)
Supplement: Supplementary Information [file srep28079-s1.doc]

**Supplementary Information**

**Identification of structurally closely related monosaccharide and disaccharide isomers by PMP labeling in conjunction with IM-MS/MS**

Hongmei Yang^1,2^, Lei Shi^3^, Xiaoyu Zhuang^2^, Rui Su^1^, Debin Wan^4^,*, Fengrui Song^2^, Jinying Li^3^ & Shuying Liu^1^,*

^1^Changchun University of Chinese Medicine, Changchun 130117, China

^2^Changchun Institute of Applied Chemistry, Chinese Academy of Sciences, 5625 Renmin Street, Changchun 130022, China

^3^High Temperature Reactor Holdings Co., Ltd., China Nuclear Engineering Group Co., Beijing 100037, China

^4^Department of Entomology and Comprehensive Cancer Center, University of California, Davis, CA 95616, United States

Correspondence and requests for materials should be addressed to S.Y.L. (email:syliu@ciac.ac.cn) or D.B.W. (email: dbwan@ucdavis.edu)

Keywords: Ion-mobility, Tandem mass spectrometry, Monosaccharide and disaccharide isomers, 1-Phenyl-3-methyl-5-pyrazolone

**Derivatization Procedures.** The standard disaccharides (0.34 mg) were brought to reaction by adding 200 μL labeling reagent solution, respectively. Then each mixture was heated at 70 °C for 120 min in a sealed vial. After the reactions, the solutions were centrifuged at 10,000 rpm for 10 min to remove sediments. Then, the supernatant was dried in nitrogen stream to remove the excess ammonia. The residue was dissolved with 1 mL ultrapure water and extracted five times with chloroform to detach the excess PMP. Finally, the aqueous layer was mixed with an equal volume of methanol for analysis. To obtain PHN derivatives, phenylhydrazine (5 μL) was added to a water solution of disaccharide (50 μL; 2.5 μM) and the mixture was incubated for 1h at 80 °C with frequent mixing. Then the samples were extracted with ethylacetate to remove the excess phenylhydrazine. The ethylacetate layer was pipetted out to another tube and washed with water (3 × 100 μL). The water fractions were mixed together and diluted with an equal volume of methanol for analysis.

**Figure legends**

**Figure S1:** Overall mobility spectra of the 10 disaccharide isomers and 4 monosaccharide isomers collected using the Synapt G2 TWIMS instrument. All mobility spectra of the disaccharides and monosaccharides were extracted for sodiated ions [M + Na]^+^ at *m/z* 365.11 and 203.05, respectively. The arrival times are from three individual measurements, and deviation is ±0.01 ms.

**Figure S2:** A generalized scheme for the reaction of carbohydrates with (a) PMP and (b) PHN in this study.

**Figure S3:** PMP and PHN derivatized mass spectra of representative carbohydrates. (a) MS of PMP-isomaltose derivative, (b) MS^2^ of PMP-isomaltose derivative, (c) MS of PMP-mannose derivative, (d) MS of PHN-isomaltose derivative, (e) MS^2^ of PHN-isomaltose derivative and (f) MS of PHN-mannose derivative.

**Figure S4:** IM mass spectrum of (a) protonated monoPMP-laminaribiose derivative and (b) sodiated laminaribiose standard.

**Figure S5:** Overall mobility spectra of the 9 PHN-derivatized disaccharide isomers and 3 PMP-derivatized monosaccharide isomers collected using the Synapt G2 TWIMS instrument. All mobility spectra of the disaccharides and monosaccharides were extracted for sodiated ions [M + Na]^+^ at *m/z* 453.16 and 291.09, respectively. The saccharides in red can be differentiated from their corresponding isomers.

**Figure S6:** IM mass spectrum of protonated maltotetraose.

**Figure S7:** Overall mobility spectra of the 10 disaccharide isomers collected using the Synapt G2 TWIMS instrument. All mobility spectra of the disaccharides were extracted for potassium adduct ions [M + K]^+^ at *m/z* 381.07.

**Fig. S1**

**Fig. S2**

**Fig. S3**

**Fig. S4**

**Fig. S5**

**Fig. S6**

**Fig. S7**

**Table S1:** CCSs (**Å^2^**) for the protonated and sodiated monoPMP derivatives of the six disaccharide isomers (n=16).

| **disaccharides** | | **CCS_[monoPMP derivatives+H]_^+^** | **CCS_[monoPMP derivatives+Na]_^+^** |
| --- | --- | --- | --- |
| kojibiose | 142.58±0.3 | | 148.98±0.5 |
| nigerose | 144.79±0.4 | | 144.01±0.4 |
| lactose | 144.43±0.3 | | 148.83±0.2 |
| sophorose | 137.69±0.2 | | 144.92±0.3 |
| cellobiose | 145.89±0.3 | | 151.52±0.4 |
| gentiobiose | 143.80±0.2 | | 151.10±0.3 |

**Table S2:** CCSs (**Å^2^**) for the potassium adduct ions of the ten disaccharide isomers (n=16).

| **disaccharides** | **CCS_[disaccharides+K]_^+^** |
| --- | --- |
| fructose | 111.2±0.2 |
| lactose | 114.1±0.1 |
| kojibiose  nigerose | 116.0±0.2  115.6±0.4 |
| maltose  isomaltose | 116.5±0.3  114.5±0.3 |
| sophorose | 115.8±0.3 |
| laminaribiose  cellobiose | 116.0±0.7  114.9±0.4 |
| gentiobiose | 114.6±0.6 |
